# Where do we go from here? – Opportunities and barriers to the career development of trial managers: a survey of UK-based trial management professionals

**DOI:** 10.1186/s13063-020-04316-z

**Published:** 2020-05-06

**Authors:** Eleanor Mitchell, Kirsteen Goodman, Suzanne Hartley, Helen Hickey, Alison M. McDonald, Helen M. Meadows, Shelley Rhodes, Jodi Taylor, Natalie Wakefield, Barbara Farrell, on behalf of the UK Trial Managers’ Network Executive Group

**Affiliations:** 1 Nottingham Clinical Trials Unit, University of Nottingham, University Park, Nottingham, NG7 2RD UK; 2 Nursing, Midwifery and Allied Health Professions Research Unit, Glasgow Caledonian University, Cowcaddens Road, Glasgow, G4 0BA UK; 3 Clinical Trials Research Unit, Leeds Institute of Clinical Trials Research, University of Leeds, Leeds, LS2 9JT UK; 4 Liverpool Clinical Trials Centre, University of Liverpool, a member of the Liverpool Health Partners, Liverpool, L69 3BX UK; 5 Centre for Healthcare Randomised Trials (CHaRT) Health Services Research Unit, University of Aberdeen, Health Sciences Building, Foresterhill, Aberdeen, AB25 2ZD UK; 6 Cancer Research UK & UCL Cancer Trials Centre & Institute of Clinical Trials & Methodology, UCL, 90 Tottenham Court Rd, London, UK; 7 Exeter Clinical Trials Unit (ExeCTU), University of Exeter, St Luke’s Campus, Exeter, Devon, EX1 2LU UK; 8 Bristol Randomised Trials Collaboration, Bristol Trials Centre, University of Bristol, 39 Whatley Road, Bristol, BS8 2PS UK; 9 National Perinatal Epidemiology Unit, Clinical Trials Unit, University of Oxford, Richard Doll Building, Headington, Oxford, OX3 7LF UK

**Keywords:** Clinical trial, Trial management, Trial manager, Career development, Survey, UKTMN, Project management

## Abstract

**Background:**

Clinical trials commonly have a dedicated trial manager and effective trial management is essential to the successful delivery of high-quality trials. Trial managers have diverse experience and currently there is no standardised structured career pathway. The UK Trial Managers’ Network (UKTMN) surveyed its members to understand what is important to them with respect to career development since this would be important in the development of any initiative intended to develop a skilled workforce.

**Methods:**

We conducted an online survey of UKTMN members, who are trial management professionals, working on academic-led trials in the UK. Members were asked what they perceive as opportunities and barriers to career development. Two reminders were sent to facilitate completion of the survey, and responders were offered the opportunity to enter a prize draw for waived fees at the UKTMN annual meeting. Data were analysed descriptively by using Stata (version 15.1), and free-text responses were reviewed for themes.

**Results:**

The survey was sent to 819 UKTMN members; 433 responses were received, although 13 were from non-UKTMN members; thus 420 respondents' data were included in analyses. Respondents were representative of UKTMN membership; however, more responses were received by trial managers based in registered clinical trials units (CTUs). The top three opportunities for career development were (i) training, (ii) helping design trials and (iii) undertaking relevant qualifications. The top three barriers were (i) funding, (ii) few opportunities to get involved in development activities aside from managing a trial and (iii) unclear organisational career pathway. Almost all respondents (401/420, 95.4%) considered career development either very or quite important. Although all respondents had a day-to-day role in managing trials, there was huge disparity between job titles.

**Conclusion:**

Career development is important to trial managers yet there is a lack of a structured pathway. The enablers and disablers to career development for trial managers should be clearly considered by the clinical trial community and, in particular, employers, sponsors and funders in order to develop a highly skilled workforce of trial managers, who are key to the delivery of trials.

## Background

Clinical trials are considered the gold standard for testing health-care interventions in patients. Not only do clinical trials need to be led by a chief investigator, but these complex projects require expert project management and it is commonplace for trials to appoint a dedicated person to manage the trial. Effective trial management has been shown to be essential to the successful delivery of high-quality trials [1], and the Strategies for Trial Enrolment and Participation Study (STEPS), which investigated strategies to improve recruitment into randomised trials, showed that the appointment of a dedicated trial manager was one factor in trials that recruited more successfully [2]. Indeed, UK major public funding bodies such as the National Institute for Health Research (NIHR) recommend the appointment of a dedicated trial manager and send a general trial manager job description at the time of project activation. Treweek and Littleford acknowledged that once clinical trial funding was awarded, the most important members of the team are “not the professors and investigators but the trial managers” who are key to delivering the goal [3], and Beaumont et al. recently wrote that having an “expert” trial manager, who can overcome operational challenges, is often the difference between success and failure of a clinical trial [4]. Over 20 years ago, one of the UK’s largest funders of clinical trials at the time, the Medical Research Council (MRC), commissioned the development of the Trial Managers’ Network in response to the failure of academic-led trials to deliver on time and budget and a lack of training identified for staff who were responsible for managing clinical trials. The network, now known as the UK Trial Managers’ Network (UKTMN), aims to facilitate the development and support of trial managers across the UK [5] and has long argued that there is a lack of recognition of the role of trial manager, particularly at an organisational level, and an absence of a structured career pathway. Trial managers come from a diverse range of backgrounds with no recognised career pathway and so usually learn “on the job” [1], which the authors acknowledge is an important part of career development though also recognise that this could be enhanced by technical and theoretical knowledge to underpin it. Recently, several publications have acknowledged a lack of career development pathways for trial managers [4, 6, 7], and Beaumont et al. recognised that whilst some progress has been made in the development of academic career pathways in some universities, not all trial managers may wish to pursue this and therefore alternative pathways should be considered. From our own experience across several organisations and discussions with UKTMN members, we find that where pathways do exist, there is disparity across organisations.

The UKTMN Executive Group, responsible for the strategic direction of the network, surveyed its membership (over 800 members) to understand what is important to them with respect to their own career development since this would be important for any initiative aimed at improving the development and retention of a skilled workforce. For the purpose of this article, a trial management professional is someone who has day-to-day responsibility for the management of operational aspects of an academic-led clinical trial or other high-quality clinical study, although the authors acknowledge that there is significant disparity between job titles across the UK. For example, a trial coordinator in one organisation could have overall responsibility for a clinical trial whereas in other organisations the role associated with this job title is a supportive one. UKTMN members are based in a variety of organisations, including universities, clinical trials units (CTUs) and National Health Service (NHS) trusts.

## Methods

We conducted an open survey of UKTMN members. Survey questions included a mixture of quantitative and free-text responses (Additional file 1). The survey was built using Jisc Online Surveys^©^, and prior to circulation a draft version of the survey was user-tested by six trial managers, all from a trial management background, in departments where authors were based. A link was circulated to all members in June 2019 and open for 20 calendar days, and two reminders were circulated during this time. Survey questions and response categories were agreed by the authors. Adaptive questioning was used when required to increase the simplicity of completion, and participants were able to review and edit their responses prior to final submission. Completion of the survey was voluntary, and consent was assumed by completion of the survey.

To encourage completion of the survey, all participants were given the option of providing their email address and being entered into a prize draw to have their delegate fees waived at the UKTMN annual meeting held later in 2019. Participants’ contact details were not linked to other survey data when analysed and were stored in the password-protected online survey. For questions relating to career development opportunities that are considered important, participants were asked to rate each opportunity from 0 to 10 (0 being not important, 10 being the most important). These data are presented as mean (standard deviation [SD]) scores. From a pre-populated list defined by the authors, participants were also asked to state which issues they perceived as barriers to career development. For both opportunities and barriers to career development, analyses were undertaken for all responses and associations that were felt to potentially influence opinion: length of time in a trial management role, salary scale (used as a proxy for level of experience given the known disparity between job titles across organisations), whether participants had previously been promoted, whether they were currently based in a UK Clinical Research Collaboration (UKCRC)-registered CTU and, for those employed within an academic setting, whether they were employed on a professional/managerial or research contract. Descriptive analyses were undertaken by using Stata (version 15.1), and free-text responses were reviewed for themes by using NVivo (version 12.1).

## Results

**Fig. 1:**
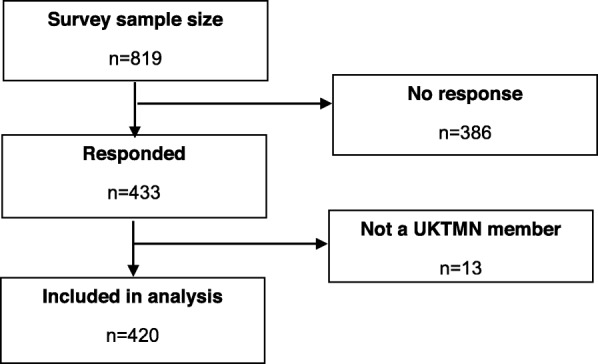
Flowchart

The survey was sent to 819 UKTMN members and 433 responses were received. Thirteen responses were received by non-members of UKTMN and this was possibly due to the fact that the survey link was forwarded by members; their data were not included in analyses and thus data are presented on 420 participants (Fig. 1).

Limited data are collected at the time of applying for UKTMN membership; however, some comparisons between characteristics of UKTMN members and those of survey respondents could be made: characteristics of survey respondents reflect that of the overall UKTMN membership for geographical spread across the UK and job titles, although there were slightly more responses from respondents who identified as a senior trial manager (93/420, 22.1%) compared with UKTMN membership (109/819, 13.3%) (Table 1).

**Table 1:** Characteristics of survey respondents

Characteristic	n = 420 (%)	Overall UKTMN membership n = 819 (%)
**Gender**		
Female	364 (86.7)	Data unavailable
Male	53 (12.6)	
Prefer not to say	3 (0.7)	
**Based in a UKCRC-registered CTU**		
Yes	317 (75.5)	464 (56.7)
No	103 (24.5)	355 (43.3)
**Location of CTU,** n = 317		
London	49 (15.5)	145 (17.7)
West Midlands	44 (13.9)	87 (10.6)
Scotland	38 (12)	86 (10.5)
Oxfordshire	35 (11)	129 (15.8)
Yorkshire and the Humber	29 (9.2)	66 (8.1)
North West	29 (9.2)	66 (8.1)
South West	22 (6.9)	73 (8.9)
East Midlands	17 (5.4)	41 (5.0)
South East	15 (4.7)	34^a^ (4.2)
Wales	14 (4.4)	28 (3.4)
East of England	14 (4.4)	42 (5.1)
South Central	7 (2.2)	34^a^ (4.2)
Northern Ireland	3 (1.0)	6 (0.7)
North East	1 (0.3)	16 (2.0)
**Employment status**		
Full-time	313 (74.7)	Data unavailable
Part-time	106 (25.3)	
Missing	1 (0.2)	
**Organisation type**		
University	356 (84.8)	729 (89.0)
NHS	60 (14.3)	86 (10.5)
Charity/Not for profit	4 (0.1)	4 (0.5)
**NHS grade/pathway,** n = 60		
Administrative/clerical	35 (58.3)	Data unavailable
Managerial	19 (31.7)	
Clinical	1 (1.7)	
Nursing/Health-care professional	4 (6.7)	
Research	1 (1.7)	
**Academic grade/pathway,** n = 356		
Professional/Administrative/Managerial/Academic-related	267 (75.1)	Data unavailable
Research	67 (18.9)	
Not known	20 (5.1)	
Academic/Academic-related (research)	2 (0.5)	
**Length of time in a trial management role**		
<1 year	32 (7.6)	Data unavailable
1–2 years	66 (15.7)	
3–5 years	126 (30)	
6–10 years	89 (21.2)	
>10 years	107 (25.5)	
**Funding for current position**		
Grant/research funding	278 (66.2)	Data unavailable
Combination of core/grant funding	77 (18.3)	
Core-funded	41 (9.8)	
Don’t know	22 (5.2)	
Institute-funding (based in a charity)	1 (0.2)	
No salary – honorary research fellow	1 (0.2)	
**Highest educational qualification**		
PhD	111 (26.4)	Data unavailable
Masters or other post-graduate qualification	138 (32.9)	
Undergraduate degree	147 (35)	
A levels or equivalent	16 (3.8)	
General Certificates of Secondary Education (GCSEs) or equivalent	6 (1.4)	
Vocational qualifications	1 (0.2)	
None of the above	1 (0.2)	
**Current job title**		
Trial manager	176 (41.9)	380 (46.4)
Senior trial manager/team lead	93 (22.1)	109 (13.3)
Trial coordinator	77 (18.3)	174 (21.2)
Trial administrator/assistant	9 (2.1)	22 (2.7)
Other	65 (15.5)	134 (16.4)
*Project manager*	*11*	
*Operations manager*	*11*	
*Research assistant/associate/fellow*	*11*	
*Assistant/associate trial manager*	*6*	
*Senior trial coordinator*	*6*	
*Senior research fellow/associate*	*4*	
*Programme manager*	*3*	
*Data manager*	*3*	
*Head of trial management*	*2*	
*Assistant professor of clinical trials*	*1*	
*Compliance manager*	*1*	
*Research facilitator*	*1*	
*Senior research manager*	*1*	
*Senior research midwife*	*1*	
*Trial master file manager*	*1*	
*Trial director*	*1*	
*Trial management director*	*1*	
**Current salary range (full-time equivalent)**		
<£18,000	1 (0.2)	
£18,000–£21,999	1 (0.2)	
£22,000–£24,999	8 (1.9)	
£25,000–£26,999	17 (4.1)	
£27,000–£31,999	70 (16.7)	
£32,000–£34,999	65 (15.5)	
£35,000–£39,999	114 (27.1)	
£40,000–£43,999	67 (16.0)	
>£44,000	61 (14.5)	
Prefer not to say	16 (3.8)	
**Ever experienced a promotion/progression to more senior role**		
Yes	224 (53.3)	Data unavailable
No	196 (46.7)	
**Reason for no experience of promotion/progression,** n = 196		
No career pathway in my organisation	66 (33.7)	Data unavailable
Not in role long enough for this to be relevant	62 (31.6)	
Never been given the opportunity	50 (25.5)	
Not wanted to	18 (9.2)	
**If promoted/progressed, how this happened,** n = 224		
Via application to new/vacant position outside your organisation	159 (71)	Data unavailable
Via a promotional pathway in your organisation	65 (29)	
**Importance of career development**		
Very important	276 (65.7)	Data unavailable
Quite important	125 (29.8)	
Not very important	18 (4.3)	
Not important at all	1 (0.2)	

*Abbreviations*: *CTU* clinical trials unit, *NHS* National Health Service, *UKCRC* UK Clinical Research Collaboration, *UKTMN* UK Trial Managers’ Network

^a^South East and South Central are counted together at time of UKTMN membership and therefore cannot be reported separately in this table; they are shown twice for transparency

There were also a higher number of responses from staff based in registered CTUs (317/420, 75.5%) compared with UKTMN membership (464/819, 56.7%); 356/420 (84.8%) of responders were based in universities, the remaining were based mainly in the NHS, and almost all respondents were on a non-academic pay scale. Surprisingly, of individuals based in an academic setting (i.e., university), only a quarter were on a research-type contract, and the majority were employed on a professional/administrative/managerial contract. Just under a third of participants had worked in a trial management role for between 3 and 5 years, and a further 20% had 6–10 years’ experience; 278/420 (66.2%) participants were funded via grant or research funding, 41/420 (9.8%) were core-funded and 77/420 (18.3%) were funded by a combination of the two. Almost all participants (396/420, 94.3%) were educated to at least undergraduate-degree level, 111/420 (26.4%) had a PhD and 138/420 (32.9%) had a masters or other postgraduate qualification; 176/420 (41.9%) participants referred to themselves as a trial manager, and the other participants had a wide range of job titles. Nearly one third (114/420) had a salary between £35,000 and £39,999. Just over half of participants (224/420) answered yes to the question “Have you ever experienced a promotion/progression to a more senior role”. For the 196 participants who had never progressed, the main reason stated was “no career pathway in my organisation” (66/196, 33.7%), and 62/196 (31.6%) participants stated that they had “not been in the role long enough for promotion to be relevant”. For the 66/196 respondents who had not progressed because there was no career pathway in their organisation, just under half (30/66, 45.5%) were based in a registered CTU. For participants who had progressed (224/420), 29% had done this via a promotional pathway in the organisation and the remaining 71% had done this via an application to a new or vacant position outside of the organisation; 401/420 (95.4%) considered career development either ‘very’ or ‘quite’ important.

### Opportunities

From a list of 12 potential opportunities considered important to career development, participants were asked to rate each one between 0 and 10 (0 being not important, 10 being the most important) (Table 2).

**Table 2:** Professional development opportunities

Opportunities that are considered important – mean (SD)	All	Length of time in a trial management role		Salary scale		Previous promotion		Based in a registered CTU		Academic grade/pathway^b^	
		<1–5 y	6- >10 y	<£35,000	>£35,000	Yes	No	Yes	No	Prof	Research
	n = 420	n = 224	n = 196	n = 162^**a**^	n = 242^**a**^	n = 224	n = 196	n = 317	n = 103	n = 267	N = 67
To attend relevant training courses	8.3 (1.8)	8.4 (1.8)	8.2 (1.9)	8.4 (1.8)	8.3 (1.8)	8.3 (1.9)	8.4 (1.8)	8.4 (1.8)	8.1 (1.9)	8.3 (1.9)	8.3 (1.8)
To help in the design of trials	7.7 (2.4)	7.5 (2.5)	8.0 (2.2)	7.4 (2.6)	7.9 (2.2)	7.8 (2.3)	7.6 (2.4)	7.8 (2.3)	7.4 (2.6)	7.7 (2.4)	8.2 (1.9)
To undertake qualifications applicable to trial management	7.5 (2.6)	7.8 (2.5)	7.2 (2.7)	7.9 (2.5)	7.3 (2.5)	7.3 (2.8)	7.8 (2.4)	7.4 (2.7)	7.8 (2.3)	7.5 (2.7)	7.7 (2.2)
To contribute to academic writing and publication	7.3 (2.7)	7.1 (2.7)	7.5 (2.6)	6.9 (2.8)	7.4 (2.6)	7.3 (2.7)	7.3 (2.7)	7.4 (2.6)	6.8 (2.9)	7.2 (2.7)	8.6 (1.7)
To join committees/ groups related to trials	7.0 (2.9)	6.9 (2.5)	7.2 (2.4)	6.8 (2.6)	7.2 (2.3)	7.0 (2.5)	7.1 (2.4)	7.0 (2.4)	7.0 (2.6)	7.0 (2.5)	7.2 (2.1)
To assist with obtaining funding for trials	7.0 (2.5)	6.6 (2.9)	7.4 (2.8)	6.5 (2.8)	7.3 (2.8)	7.3 (2.7)	6.6 (3.0)	7.1 (2.8)	6.5 (3.0)	7.0 (2.9)	7.8 (2.4)
To work on more complex trials	6.5 (2.6)	6.6 (2.5)	6.4 (2.8)	6.5 (2.5)	6.5 (2.5)	6.5 (2.8)	6.6 (2.5)	6.6 (2.6)	6.5 (2.7)	6.5 (2.8)	6.5 (2.3)
To work in a variety of clinical areas	6.4 (2.7)	6.6 (2.5)	6.2 (2.8)	6.9 (2.4)	6.1 (2.8)	6.3 (2.7)	6.6 (2.6)	6.5 (2.7)	6.1 (2.6)	6.4 (2.7)	6.1 (2.9)
To contribute to methodological studies/SWATs	6.1 (3.0)	5.8 (2.9)	6.5 (3.0)	5.9 (3.0)	6.3 (3.0)	6.2 (3.0)	6.0 (3.0)	6.3 (2.9)	5.7 (3.0)	6.3 (3.0)	6.7 (2.7)
To present work at conferences	6.1 (3.0)	6.0 (2.9)	6.1 (3.1)	5.7 (3.1)	6.2 (2.9)	6.2 (2.9)	6.2 (2.9)	6.1 (3.0)	5.9 (3.0)	6.0 (3.0)	7.1 (2.8)
To work on trials outside the UK	5.5 (3.2)	5.4 (3.3)	5.5 (3.2)	5.7 (3.3)	5.3 (3.2)	5.6 (3.2)	5.3 (3.3)	5.5 (3.2)	5.3 (3.4)	5.8 (3.2)	5.0 (3.3)
to work on larger (sites and participants) trials	5.4 (2.9)	5.7 (2.7)	5.1 (3.0)	5.7 (2.8)	5.2 (3.0)	5.3 (3.0)	5.6 (2.8)	5.3 (2.9)	5.7 (3.0)	5.5 (2.9)	5.1 (2.7)

*Abbreviations*: *CTU* clinical trials unit, *SD* standard deviation, *SWAT* study within a trial

^a^ For salary scale, the 16 respondents who answered “prefer not to say” were not included in this analysis

^b^ For academic grade/pathway, only responses from respondents based in a University are included. Prof = “Professional/Administrative/Managerial/Academic-related”. Twenty respondents are not included since contract type was unknown

The top three perceived most important opportunities were (1) to attend relevant training courses (mean 8.3, SD 1.8), (2) to help in the design of trials (mean 7.7, SD 2.4) and (3) to undertake relevant qualifications applicable to trial management (mean 7.5, SD 2.6). The most important opportunity for almost all groups, irrespective of possible associations previously described, was “to attend relevant training courses”. The top three opportunities by the characteristics described above are shown in Table 3. Participants were also asked to state any other opportunities that they considered important with respect to career development; in total, there were 35 responses, many of which were covered by the list of opportunities given, although several responses stated that they would like the opportunity to develop leadership and line management responsibilities.

**Table 3:** Top three perceived enablers of career development

	Top three professional development opportunities		
	1	2	3
**All respondents**	Training	Trial design	Qualifications
**Length of time in a trial management role**			
<1–5 years	Training	Qualifications	Trial design
6- >10 years	Training	Trial design	Qualifications
**Salary scale**			
<£35,000	Training	Qualifications	Trial design
>£35,000	Training	Trial design	Academic writing
**Previously promoted**			
Yes	Training	Trial design	Qualifications, academic writing, assist with funding
No	Training	Qualifications	Trial design
**Based in a registered clinical trials unit**			
Yes	Training	Trial design	Qualifications, academic writing
No	Training	Qualifications	Trial design
**Academic grade/pathway**			
Professional	Training	Trial design	Qualifications
Research	Academic writing	Training	Trial design

Full wording for each opportunity is as follows: “Training” – to attend relevant training courses; “Trial design” – to help in the design of trials; “Qualifications” – to undertake qualifications applicable to trial management; “Academic writing” – to contribute to academic writing and publications; “Assist with funding” – to assist with obtaining funding for trials

### Barriers

Participants were asked to select perceived barriers to career development from a list of nine with the option of giving additional barriers not included in the list (Table 4). The top three perceived barriers were “funding” (256/420, 61%), “few opportunities to get involved in other activities aside from managing a clinical trial(s)” (249/420, 59.3%) and “unclear career pathway within the organisation” (247/420, 58.8%).

**Table 4:** Perceived barriers to professional development

Perceived barriers to career development	All responses	Length of time in a trial management role		Salary scale		Previous promotion		Based in a registered CTU		Academic grade/pathway^b^	
		<1–5 years	6- >10 years	<£35,000	>£35,000	Yes	No	Yes	No	Prof	Research
	n = 420 (%)	n = 224 (%)	n = 196 (%)	n = 162^a^ (%)	n = 242^a^ (%)	n = 224(%)	n = 196 (%)	n = 317	n = 103	n = 267	n = 67
Funding	256 (61.0)	132 (58.9)	124 (63.3)	103 (63.6)	141 (58.3)	136 (60.7)	120 (61.2)	186 (58.7)	70 (67.8)	163 (61.1)	35 (52.2)
Few opportunities aside managing a trial(s)	249 (59.3)	123 (54.9)	126 (64.3)	98 (60.5)	141 (58.3)	138 (61.7)	111 (56.6)	192 (60.6)	57 (55.3)	161 (60.3)	39 (58.2)
Unclear career pathway in organisation	247 (58.8)	122 (54.5)	125 (63.8)	84 (51.9)	151 (62.4)	124 (54.9)	124 (63.3)	170 (53.6)	77 (74.8)	154 (57.7)	36 (53.7)
Recognition of role	237 (56.4)	106 (47.3)	131 (66.8)	84 (51.9)	142 (58.7)	135 (60.3)	102 (52.0)	178 (56.2)	59 (57.3)	156 (58.4)	36 (53.7)
Lack of time	223 (53.1)	114 (50.9)	109 (55.6)	84 (51.9)	129 (53.3)	136 (60.7)	87 (44.4)	178 (56.2)	45 (43.7)	142 (53.2)	39 (58.2)
Training	155 (37.0)	94 (42.0)	61 (31.1)	82 (50.6)	66 (27.3)	77 (34.4)	78 (39.8)	113 (35.7)	42 (40.8)	99 (37.1)	20 (29.9)
Geographical location	63 (15)	31 (13.8)	32 (16.3)	22 (13.6)	37 (15.3)	36 (16.1)	27 (13.8)	45 (14.2)	18 (17.5)	42 (15.7)	5 (7.5)
Size of organisation	54 (12.9)	32 (14.3)	22 (11.2)	25 (15.4)	28 (11.6)	25 (11.2)	29 (14.9)	37 (11.7)	17 (16.5)	27 (10.1)	12 (17.9)
No barriers perceived	10 (2.4)	6 (2.7)	4 (2.0)	3 (1.9)	6 (2.5)	4 (1.8)	6 (3.1)	7 (2.2)	3 (2.9)	8 (3.0)	1 (1.5)

*Abbreviation*: *CTU* clinical trials unit

^a^ For salary scale, the 16 respondents who answered “prefer not to say” were not included in this analysis

^b^ For academic grade/pathway, only responses from respondents based in a university are included. Prof = “Professional/Administrative/Managerial/Academic-related”. Twenty respondents are not included since contract type was unknown

The top three barriers to career development, by the characteristics previously described, are shown in Table 5. Participants were asked to state any other barriers perceived, not included in the pre-populated list. These included; (i) fixed term contracts, (ii) line managers' insistence on the need to focus on "delivery of trials", (iii) an assumption by the academic community that academic qualifications are needed to progress when experience is often just as valuable and (iv) the job title "trial manager" can imply an administrative rather than professional to many.

**Table 5:** Top three perceived barriers to career development

	Top three perceived barriers to career development		
	1	2	3
**All respondents**	Funding	Few opportunities	Unclear career pathway
**Length of time in a trial management role**			
<1–5 years	Funding	Few opportunities	Unclear career pathway
6- >10 years	Recognition	Few opportunities	Unclear career pathway
**Salary scale**			
<£35,000	Funding	Few opportunities	Unclear career pathway, recognition, lack of time
>£35,000	Unclear career pathway	Recognition	Funding, few opportunities
**Previously promoted**			
Yes	Few opportunities	Funding, lack of time	Recognition
No	Unclear career pathway	Funding	Few opportunities
**Based in a registered clinical trials unit**			
Yes	Few opportunities	Funding	Recognition, lack of time
No	Unclear career pathway	Funding	Recognition
**Academic grade/pathway**			
Professional	Funding	Few opportunities	Recognition
Research	Few opportunities, lack of time	Recognition, unclear career pathway	Funding

Full wording for each barrier is as follows: “Funding” – funding; “Few opportunities” – few opportunities aside managing a trial(s); “unclear career pathway” - unclear career pathway in organisation; “recognition” – recognition of role; “lack of time” – lack of time

Participants were asked to report how well supported they felt, with respect to professional development, by their line manager, department/unit (within the organisation) and the organisation itself. Nearly half of participants (194/420, 46.2%) felt “very well supported” by their line manager, although only a quarter (103/420, 24.5%) felt the same about their department/unit and less for their organisation (67/420, 16.0%). 115/420 (27.4%) participants provided free-text comments on other areas that they felt related to career development for trial managers and that had not been covered by previous questions. The main themes identified from free-text responses were (1) lack of recognition, understanding and value of the role of trial manager, (2) fixed/short-term contracts and funding, (3) lack of training and clear career pathway, (4) disparity between organisations and (5) lack of time and capacity to focus on career development. Comments reflected the quantitative data and are shown in Table 6.

**Table 6:** Themes identified from free-text comments

Theme	Example quotes
Lack of recognition, understanding and value of the role of trial manager	“Trial managers are seen as less important than academics in the university setting”.
	“I don’t think trial management is seen as a professional career in the academic community”.
	“There is a significant problem with recognising the contribution a trial manager makes to the team”.
	“I was once called a glorified administrator by an academic”.
Fixed/short-term contracts and funding	“Short-term funding is the key barrier to career development”.
	“Although I work in a large registered CTU, I do not feel that the training is given to support the move to the next level within the unit”.
Lack of training and clear career pathway	“There is no guidance out there about what training would be best for career progression”.
	“The role feels very much like a dead-end job with no official training or career pathway”.
	“I would like to be able to send the staff on more external development opportunities, but it is difficult to see where that funding would come from”.
Disparity between organisations	“Having worked on trials in different organisations and worked with colleagues who have also worked on trials in other trials units I think there are discrepancies across the country in terms of grading of trial manager and senior trial manager positions”.
	“Huge disparity between organisations on how the role is treated and valued”.
	“Lack of consistency between units - the name of roles is not consistent”.
Lack of time and capacity to focus on career development	“Your whole time is taken up with the demanding work of keeping your trial running”.
	“I think time and money are the biggest factors affecting career development”.
	“There is lack of funding to provide time to develop outside the immediate delivery of the trial”.

*Abbreviation*: *CTU* clinical trials unit

## Discussion

### Relevance to trial management professionals within the UK

Although publications in the past have highlighted the importance of career development for trial management professionals, this is the first published survey (to our knowledge) to demonstrate what is important to trial management professionals with respect to their own career development. The views of trial management professionals are important to consider by organisations involved in clinical trials when developing any initiatives to improve the development, retention and remuneration of their staff. Our response rate was consistent with other surveys in organisational research [8]. Although we recognise that the response rate of 51.3% could lead to non-response bias, 420 participants were included in the analyses and, other than a higher response from trial management professionals based in registered CTUs than overall, UKTMN members who responded to the survey were generally representative of UKTMN members. Participants had a range of experience, varying between less than 1 year to more than 10 years, and there was roughly an equal split between participants who had experienced a previous promotion to a more senior role and those who had not (53% vs. 46%), demonstrating the relevance of these survey results to trial managers based in the UK. There is clear disparity between organisations with respect to the job titles of trial management professionals, which could be a contributing factor to the development of a structured career pathway. The topic of career development is clearly important to trial management professionals, as demonstrated by the fact that almost all (401/420, 95.5%) participants rated career development as either ‘very’ or ‘quite’ important to them.

### Opportunities for career development of trial management professionals

Our data have shown that attending relevant training courses is the most important opportunity for trial management professionals when they are considering career development. There were only minor differences in sub-analyses when considering the associations of length of time in a trial management role, their salary scale, whether they had previously been promoted/progressed or whether they were based in a registered CTU. This demonstrates a clear opportunity for universities, NHS trusts and CTUs to ensure adequate investment in training for trial management professionals in order for them to manage clinical trials to the high quality required. Large funders such as the NIHR are offering many training opportunities through initiatives such as the NIHR Academy, although almost all are focussed on training for NHS health professionals [9] rather than operational staff who coordinate clinical trials located mostly in a university setting. Some differences were seen between trial management professionals employed on a research contract rather than a professional/managerial contract. Unsurprisingly, those employed on a research contract considered the most important opportunity to be involved in academic writing (mean score 8.6/10). Attending conferences was also placed higher in those on a research contract compared with those on a professional/managerial contract (7.1/10 vs. 6/10). Both of these are consistent with common tasks an academic researcher would be involved in, so understandably trial management professionals employed on a research contract would see merit in having opportunities to develop in these areas. Participants also rated helping with the design of trials highly (second most important opportunity for all participants; mean score overall 7.7/10), and trial management professionals who had been in their role for more than 6 years scored this slightly higher than those in the role for less than 5 years. This is consistent with the fact that trial management professionals who are more highly paid also scored the opportunity to assist with obtaining funding for trials highly (mean score 7.3/10). This could suggest that the desire to input into trial design and funding applications comes with more trial management experience, potentially having a deeper understanding of the importance of trial delivery and having seen design issues leading to difficulties with the conduct of the trial further along in the trial life cycle. This is consistent with a recent commentary by Kelly et al. [7], who suggest that lead investigators should invite senior operational staff to be co-applicants on trial grant applications to prevent such issues from arising. Investigators and academic staff within CTUs should include experienced trial management professionals in the design stage of their trials as they can add value and make an important contribution. In addition, this could facilitate the career development and job satisfaction of the experienced trial managers. Participants considered obtaining relevant qualifications applicable to trial management as important (all participants mean score 7.5/10, third most important opportunity) and this was seen as slightly more important for trial management professionals with less experience (<1–5 years) (mean score 7.8/10, second highest opportunity) and those based outside of registered CTUs (mean score 7.8/10, second highest opportunity). Although there are opportunities to obtain qualifications such as a clinical trials MSc offered by various universities, the value of such qualifications is unclear since they do not link to a specific career pathway. In addition, although employability following completion of one such MSc in clinical trials, at University College London, has increased and led to promotion (Helen M. Meadows, personal communication), it can be difficult for trial managers to attend such courses because of some of the barriers already outlined.

### Barriers to career development

Lack of funding and fixed-term contracts were clearly identified as barriers to career development; 61% of all participants stated that this was an issue and there was little difference in perception between trial management professionals with less or more experience. In an environment where funding for these types of roles is often project-based and for a time-limited period, this is difficult, and universities and NHS organisations should consider how trial managers may be given the same opportunities as tenured staff. Participants also identified that they are given few opportunities to get involved in activities outside of the day-to-day running of their clinical trial and this perception was greater amongst trial management professionals with more than 6 years’ experience. Investigators and CTUs could consider how trial management professionals work together, possibly in teams (as is the case in many CTUs), to ensure adequate and appropriate cover for their clinical trials, whilst balancing the development needs of individuals to allow them opportunities to get involved in other activities outside of their trial (e.g., training, trial design, and funding applications). It was no surprise to the authors that an unclear career pathway within an organisation was identified as a barrier to career development by nearly 60% of participants (slightly higher for trial management professionals with more than 6 years’ experience); this reflects an area that the UKTMN has highlighted since its launch over 20 years ago. For trial managers based outside of CTUs, this is clearly a bigger barrier; almost three quarters of participants (74.8%) stated that this was a barrier, and amongst this sub-group, this was the number one barrier identified. This is in line with 56% of participants also identifying that ‘recognition of the role’ is a barrier to career development and that, whilst most participants felt supported by their line manager (quite likely to be someone who works within clinical trials themselves), less support at a department and organisational level was identified. A structured career pathway, where trial management is recognised as a profession in its own right by employing organisations and funders, is a long-term vision of the UKTMN. Within many universities, there are differences between promotional pathways for staff on research contracts and staff on professional/administrative contracts. Trial management professionals, employed on a research contract, could have the opportunity to apply for promotion via a standard promotions pathway for research staff. In order to achieve this promotion, they should be given opportunities to be involved in academic writing, publishing, and applying for funding opportunities. However, those employed on a professional/managerial contract often do not have the same opportunity and would require their position to be re-graded. In universities, staff who are managing trials should be recognised for their input in the same way that academic staff are. Our results show that this is even more important if you are not based within a registered CTU and may not have this kind of support. As previously recognised, without a trial manager to manage the day-to-day activities of a clinical trial, even the best designed trial could fail [1].

### Strengths and limitations

This is the first known study reporting what trial management professionals consider important with respect to their career development. The results of the survey could help facilitate and shape the development of a standardised career pathway for trial management professionals within the UK. It is also hoped that employers, sponsors and funders give careful thought to a variety of opportunities that could exist for trial management professionals wishing to develop their own careers; these may well differ depending upon the type of contract the individual is employed on. By identifying perceived barriers to career development, the organisations involved in clinical trials within the UK can see where changes are required in order to recognise the contribution that trial management professionals make. In order to design and deliver high-quality research, it is in the interest of the clinical trial community that consistency be introduced for trial management professionals. For example, funding bodies could consider providing funds for training within grants or bursaries for trial management professionals. We recognise that a limitation of this survey is that only members of the UKTMN were asked to complete this, thus introducing an element of selection bias; however, UKTMN members are geographically spread across the UK, come from a range of UK organisations and therefore are largely representative of trial management professionals in the UK. The response rate of 51.3% could have introduced some non-response bias, although every effort was made to increase the response rate, including sending several reminders directly to members, a social media presence and the incentive of a waived fee for the 2019 annual meeting upon completion of the survey, and respondents are representative of the UKTMN membership. A second limitation is that our survey did not prevent the possibility of duplicate entries from the same participant, although we acknowledged this in reminders, asking UKTMN members to complete it only if they had not done so previously. We consider the results of this survey as relevant to anyone working in clinical trials but also recognise that this is a small step in the wider national development of a structured pathway. Future research could focus on career development of trial management professionals from a multi-stakeholder and organisational perspective since ultimately a career development pathway is a higher strategic development that would require buy-in from many stakeholders across and beyond the clinical trials landscape.

## Conclusions

Career development is considered important and highly relevant to trial management professionals. The opportunities and barriers to career development identified in the survey could help investigators, organisations, CTUs and funders facilitate a structured career pathway in the future. Ensuring that the development needs of trial management professionals are met is in the interest of all stakeholders involved in clinical trials since, without their skills and expertise, high-quality successful clinical trials will not be delivered effectively.

## Supplementary information

**Additional file 1.**

## Data Availability

The datasets used and analysed during this study are available from the corresponding author upon reasonable request.

## Abbreviations

CTU: Clinical trials unit
NHS: National Health Service
NIHR: National Institute for Health Research
SD: Standard deviation
UKTMN: UK Trial Managers’ Network
