# Supplementary material for: Where do we go from here? – Opportunities and barriers to the career development of trial managers: a survey of UK-based trial management professionals
Source: Trials. 2020 May 6;21:384. doi: 10.1186/s13063-020-04316-z (PMC7203789; doi:10.1186/s13063-020-04316-z)
Supplement: Supplementary file 1 — Additional file 1. [file 13063_2020_4316_MOESM1_ESM.pdf]

# UKTMN Career Development Survey

## Page 1: Introduction

Effective trial management is essential to the delivery of high quality clinical trials, yet the career pathway for trial managers across the UK is variable; this is something that the UK Trial Managers' Network is hoping to change. Whilst we recognise the lack of career structure, we feel that in order to make progress, it is essential we understand what is important for trial management professionals with respect to their personal and professional development.

We would therefore be grateful if you could complete this short survey, which we anticipate will take 5-10 minutes to complete.  You are not required to give any personal information.

We are committed to support the development of trial management professionals and urge as many people working in a trial management role to complete the survey as possible, so that your opinions and views count. We are interested in the experiences and opinions of  trial management professionals at all levels.

All the answers you provide are entirely anonymous, and they will not and cannot be linked back to you. Please bear this in mind when answering the questions.

At the end of the survey, you will get the chance to enter a prize draw for a place at the UKTMN Annual Meeting. The contact information you give will not be associated with your survey data.

We aim to publish the results of this survey in a scientific journal and will also be submitting an abstract to the International Clinical Trials Methodology Conference, being held in Brighton in October 2019.

If you have any questions, please contact uktmn@nottingham.ac.uk

Many thanks

## Page 2: Q1

Q1. Are you currently working as a trial management professional in the UK?

- ○ Yes
- ○ No

## Page 3: Demographics 1

Q2. Are you

- Male
- Female
- Prefer not to say

Q3. Do you work within a UKCRC registered Clinical Trials Unit (CTU)?

- Yes
- No

If yes, where is your CTU based?

- East Midlands
- East of England
- London
- North East
- North West
- Northern Ireland
- Oxfordshire
- Scotland
- South Central
- South East
- South West
- Wales
- West Midlands
- Yorkshire and the Humber

Q4. Are you a member of the UKTMN?

- Yes
- No

Do you consider yourself full-time or part-time?

Q5. What kind of organisation are you employed by?

- University
- NHS
- Other

If based within an NHS setting, what type of grade/pathway are you on?

- Administrative/Clerical
- Managerial
- Clinical
- Nursing/Healthcare Professional
- Other

If you selected Other, please specify:

If based within an academic setting, what type of grade/pathway are you on?

- Professional/Administrative/Managerial/Academic-related
- Research
- Not Known
- Other

If you selected Other, please specify:

If you selected Other, please specify:

Q6. How long have you worked in a Trial Management role?

- <1 year
- 1-2 years
- 3-5 years
- 6-10 years
- >10 years

## Page 4: Demographics 2

Q7. How is your **current** position funded?

- Grant/research funding
- Core-funded
- Combination of core / grant funding
- Don’t know
- Other

If you selected Other, please specify:

Q8. What is your highest educational qualification?

- PhD
- Masters or other post-graduate qualification
- Undergraduate degree
- A levels or equivalent
- GCSEs or equivalent
- Vocational qualifications
- None of the above

Q9. What is your current job title?

- Trial Administrator/Assistant
- Trial Co-ordinator
- Trial Manager
- Senior Trial Manager/Team Lead
- Other

If you selected Other, please specify:

Q10. What is your salary range? If you work part-time, please complete this for your full-time equivalent salary. (n.b. collecting salary gives us an idea of whether there is some parity across organisations between job titles and salary, all the information you give is anonymous)

- Less than £18,000
- £18,000 – £21,999
- £22,000 – £24,999

- £25,000 – £26,999
- £27,000 – £31,999
- £32,000 – £34,999
- £35,000 - £39,999
- £40,000- £43,999
- More than £44,000
- Prefer not to say

Q11. Have you ever been promoted or progressed to a more senior role whilst working in a trial management role?

- Yes
- No

If no, what is the reason?

- Never been given the opportunity for promotion or progression to a more senior role
- There isn’t a career pathway in my organisation that enables promotion or progression to a more senior role
- I have not wanted to be promoted or progress to a more senior role
- I have not been in my role long enough for this to be relevant

If yes, how were you promoted? *If this has occurred more than once, please respond according to your most recent promotion.*

- Via a promotional pathway within your employing organisation
- Via application to a new/vacant position within or outside your employing organisation

# Page 5: Opinions

Q12. How important is career development to you?

- ○ Very important
- ○ Quite important
- ○ Not very important
- ○ Not important at all

Q13. Professional development is not just about promotion. For each opportunity listed below, please rate the level of importance they have to you personally (at this moment in time), with 0 being "not important at all", and 10 being "extremely important".

| | 0 | 1 | 2 | 3 | 4 | 5 | 6 | 7 | 8 | 9 | 10 |
|---|---|---|---|---|---|---|---|---|---|---|---|
| to work on larger (sites and participants) trials | ☐ | ☐ | ☐ | ☐ | ☐ | ☐ | ☐ | ☐ | ☐ | ☐ | ☐ |
| to work on more complex trials | ☐ | ☐ | ☐ | ☐ | ☐ | ☐ | ☐ | ☐ | ☐ | ☐ | ☐ |
| to work in a variety of clinical areas | ☐ | ☐ | ☐ | ☐ | ☐ | ☐ | ☐ | ☐ | ☐ | ☐ | ☐ |
| to work on trials outside of the UK | ☐ | ☐ | ☐ | ☐ | ☐ | ☐ | ☐ | ☐ | ☐ | ☐ | ☐ |
| to help in the design of trials | ☐ | ☐ | ☐ | ☐ | ☐ | ☐ | ☐ | ☐ | ☐ | ☐ | ☐ |
| to assist with obtaining funding for trials | ☐ | ☐ | ☐ | ☐ | ☐ | ☐ | ☐ | ☐ | ☐ | ☐ | ☐ |
| to contribute to academic writing and publication | ☐ | ☐ | ☐ | ☐ | ☐ | ☐ | ☐ | ☐ | ☐ | ☐ | ☐ |
| to contribute to methodological studies, e.g. SWATs | ☐ | ☐ | ☐ | ☐ | ☐ | ☐ | ☐ | ☐ | ☐ | ☐ | ☐ |
| to present work at conferences | ☐ | ☐ | ☐ | ☐ | ☐ | ☐ | ☐ | ☐ | ☐ | ☐ | ☐ |
| to attend relevant training courses | ☐ | ☐ | ☐ | ☐ | ☐ | ☐ | ☐ | ☐ | ☐ | ☐ | ☐ |

| | | | | | | | | | | | |
|---|---|---|---|---|---|---|---|---|---|---|---|
| to undertake relevant qualifications, applicable to trial management (e.g. post-graduate qualifications, project management, clinical trials training courses) | ☐ | ☐ | ☐ | ☐ | ☐ | ☐ | ☐ | ☐ | ☐ | ☐ | ☐ |
| to join committees/other groups (internal and external) related to clinical trials | ☐ | ☐ | ☐ | ☐ | ☐ | ☐ | ☐ | ☐ | ☐ | ☐ | ☐ |

If there are any opportunities that are important to you, and not listed in the table above, please list them here *Optional*

Q14. How well supported do you feel with respect to your professional development from the parties shown in the table?

| | Not at all supported | Not very supported | Somewhat supported | Very well supported |
|---|---|---|---|---|
| Line manager | ☐ | ☐ | ☐ | ☐ |
| Your department/Unit (within your organisation) | ☐ | ☐ | ☐ | ☐ |
| Your organisation | ☐ | ☐ | ☐ | ☐ |

## Page 6: Opinions 2

Q15. What barriers do you perceive relating to career development for trial managers? *Tick all that apply*

- ☐ Lack of time
- ☐ Few opportunities to get involved in other activities aside from managing a clinical trial(s)
- ☐ Training
- ☐ Funding
- ☐ Recognition of role
- ☐ Geographical location
- ☐ Size of organisation
- ☐ Unclear career pathway within organisation
- ☐ No barriers perceived
- ☐ Other

If you selected Other, please specify:

Q16. Do you have any general comments relating to career development for trial managers in the UK, not covered by the previous questions?

Thank you for completing the survey. Please provide your email address below if you would like to enter a prize draw for the chance to win a free place at this years UKTMN Annual Meeting, worth £130!  The meeting will take place on Tuesday 22nd October 2019 in Birmingham.  Please be aware, the email address you provide will not be associated to any survey data you have provided. Your responses to the survey are anonymous.

Please enter a valid email address.

## Page 7: Final page

The survey is now complete. Thank you for your time.

---

# Key for selection options

**5 - Do you consider yourself full-time or part-time?**

- Full-time
- Part-time

---
